# Serum MicroRNA-122 Predicts Survival in Patients with Liver Cirrhosis

**DOI:** 10.1371/journal.pone.0045652

**Published:** 2012-09-28

**Authors:** Oliver Waidmann, Verena Köberle, Friederike Brunner, Stefan Zeuzem, Albrecht Piiper, Bernd Kronenberger

**Affiliations:** Department of Medicine 1, Klinikum der Goethe-Universität, Frankfurt, Germany; University of Nebraska Medical Center, United States of America

## Abstract

**Background:**

Liver cirrhosis is associated with high morbidity and mortality. MicroRNAs (miRs) circulating in the blood are an emerging new class of biomarkers. In particular, the serum level of the liver-specific miR-122 might be a clinically useful new parameter in patients with acute or chronic liver disease.

**Aim:**

Here we investigated if the serum level of miR-122 might be a prognostic parameter in patients with liver cirrhosis.

**Methods:**

107 patients with liver cirrhosis in the test cohort and 143 patients in the validation cohort were prospectively enrolled into the present study. RNA was extracted from the sera obtained at the time of study enrollment and the level of miR-122 was assessed. Serum miR-122 levels were assessed by quantitative reverse-transcription PCR (RT-PCR) and were compared to overall survival time and to different complications of liver cirrhosis.

**Results:**

Serum miR-122 levels were reduced in patients with hepatic decompensation in comparison to patients with compensated liver disease. Patients with ascites, spontaneous bacterial peritonitis and hepatorenal syndrome had significantly lower miR-122 levels than patients without these complications. Multivariate Cox regression analysis revealed that the miR-122 serum levels were associated with survival independently from the MELD score, sex and age.

**Conclusions:**

Serum miR-122 is a new independent marker for prediction of survival of patients with liver cirrhosis.

## Introduction

End stage liver disease is associated with high morbidity and mortality. When hepatic decompensation has occurred, the morbidity increases rapidly. After the first manifestation of ascites the one year mortality increases up to 40% [1], [2]. The patients' prognosis further deteriorates upon occurrence of spontaneous bacterial peritonitis or hepatorenal syndrome. Also variceal bleeding has a high impact on the death rate (around 20%) in every episode in patients with liver cirrhosis [3]. The assessment of the risk of death in these patients is of high interest and importance to optimize the time point for organ allocation if liver transplantation is indicated [4]. Initially developed for estimating risks in patients undergoing transjugular portosystemic shunt procedure [5], the model of end stage liver disease (MELD) score has now been used for several years to attribute the risk of death for patients with liver cirrhosis, rendering it the lead parameter for allocation of livers for transplantation [6], [7]. The MELD score is also predictive for patients with complications of liver cirrhosis such as acute bleeding or hepatorenal syndrome [8], [9]. However, the MELD score has some shortcomings as it does not include all complications of liver cirrhosis, e.g. ascites or hepatic encephalopathy. Furthermore, the MELD score was developed for prediction of short term survival within 3 months. Predictivity for survival of more than 3 months is much weaker [6]. Therefore, the evaluation of new markers is an important task in patients with liver cirrhosis.

MicroRNAs (miRs), evolutionary highly conserved, small (18–25 ribonucleotides) non-coding RNAs, are involved in the regulation of virtually all cell functions [10]. They currently emerge as a new class of biomarkers [11]–[13]. Dysregulated miRs play pivotal roles in the pathogenesis of various diseases and characteristic miR pattern have been found in pathological tissues [14]. In the liver miR-122 accounts for approximately 70% of all miRs and is important for the functional state of the hepatocyte, whereas other organs express much lower amounts of this miR [15], [16]. miR-122 regulates many genes in the liver that control the cell cycle, differentiation, proliferation and apoptosis [17]. Loss of miR-122 in the liver leads to hepatic dedifferentiation with a malignant phenotype [18]. miRs, probably in large part derived from cells with damaged plasma membrane [19], also circulate in the blood in a cell-free and relatively stable form [13]. Differences in the concentration of certain miRs have been found between sera or plasma from patients with cancer, inflammatory or cardiovascular diseases, rendering cell-free blood-derived miRs a highly promising new class of disease markers [12]. However, to fully evaluate the diagnostic potential of serum- or plasma-derived miRs, further clinical studies, in particular prospective studies, are necessary.

**Table 1:** Patient characteristics.

Parameter	Test cohort	Validation cohort
Epidemiology		
Patients	107	143
Gender, male/female, n (%)	71/36 (66.4/33.6)	96/47 (67.1/32.9)
Age, years, median, SD	57±11.3	58±11.7
Etiology of liver cirrhosis		
Alcohol abuse, n (%)	60 (56.1)	65 (45.5)
Hepatitis C, n (%)	32 (29.9)	34 (23.8)
Hepatitis B, n (%)	13 (12.1)	18 (12.6)
Hepatitis D, n (%)	1 (0.9)	0 (0.0)
Primary sclerosing cholangitis, n (%)	4 (3.7)	11 (7.7)
Autoimmune hepatitis, n (%)	4 (3.7)	5 (3.5)
Non alcoholic steatohepatitis, n (%)	4 (3.7)	2 (1.4)
Alpha1-antitrypsin deficiency, n (%)	1 (0.9)	0 (0.0)
Cardiac cirrhosis, n (%)	1 (0.9)	0 (0.0)
Cryptogenic, n (%)	6 (5.6)	18 (12.6)
Budd-Chiari syndrome, n (%)	1 (0.9)	0 (0.0)
Hemochromatosis, n (%)	3 (2.8)	4 (2.8)
Primary biliary cirrhosis	0 (0.0)	3 (2.1)
Hepatocellular carcinoma, n (%)	15 (14.0)	23 (16.1)
MELD^1^, mean, SD	16±7	14±6
Laboratory results		
Sodium^2^ (mmol/l), median, SD	138±5.1	139±5.3
ALT^3^ (U/l), median, SD	30±157	33±126
AST^4^ (U/l), median, SD	55±125	50±239
GGT^5^ (U/l), median, SD	99±196	108±202
ALP^6^ (U/l), median, SD	114±82	126±94
Albumin^7^ (g/dl), median, SD	3.2±0.6	3.2±0.7
Bilirubin^8^ (mg/dl), median, SD	2.3±5.8	1.8±4.6
INR^9^, median, SD	1.48±0.48	1.31±0.36
Creatinine^10^ (mg/dl), median, SD	1.08±0.88	0.91±0.68

Abbreviations: ^1^model of end stage liver disease, ^2^normal value: 135–145 mmol/l ^3^alanine aminotransferase (normal values: female: 10–35 U/l, male: 10–50 U/l), ^4^aspartate aminotransferase (normal values: female: <35 U/l, male: <40 U/l), ^5^γ-glutamyltransferase (normal values: female: <40 U/l, male: <60 U/l), ^6^alkaline phosphatase (normal values: female: 55–105 U/l, male: 40–130 U/l), ^7^normal values: 3.5–5.2 mg/dl, ^8^normal values: <1 mg/dl, ^9^international normalized ratio, ^10^normal values: female: 0.5–0.9 mg/dl, male: 0.7–1.2 mg/dl.

**Figure 1:**
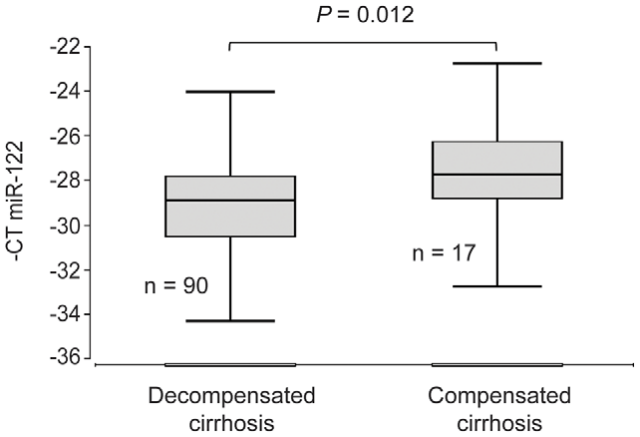
Serum miR-122 levels in cirrhotic patients with and without hepatic decompensation. The vertical lines indicate the range, the horizontal boundaries of the boxes represent the first and third quartile. The number of patients in each group is indicated in the figure.

Elevated levels of the liver-specific miR-122 have been found in sera or plasma from humans and rodents upon toxic liver injury [20]–[22] as well as in sera or plasma of patients with chronic hepatitis B or C infection [23]–[26], rendering miR-122 a promising specific marker for liver diseases. The suitability of cell-free miRs in the blood to predict patient survival of patients with liver diseases is presently unknown. To investigate if miR-122 might be a prognostic parameter in patients with liver cirrhosis, we examined miR-122 levels in sera from patients with compensated and decompensated liver cirrhosis and correlated the serum miR-122 levels with patient survival, development of complications and standard laboratory parameters in a prospective clinical study.

**Figure 2:**
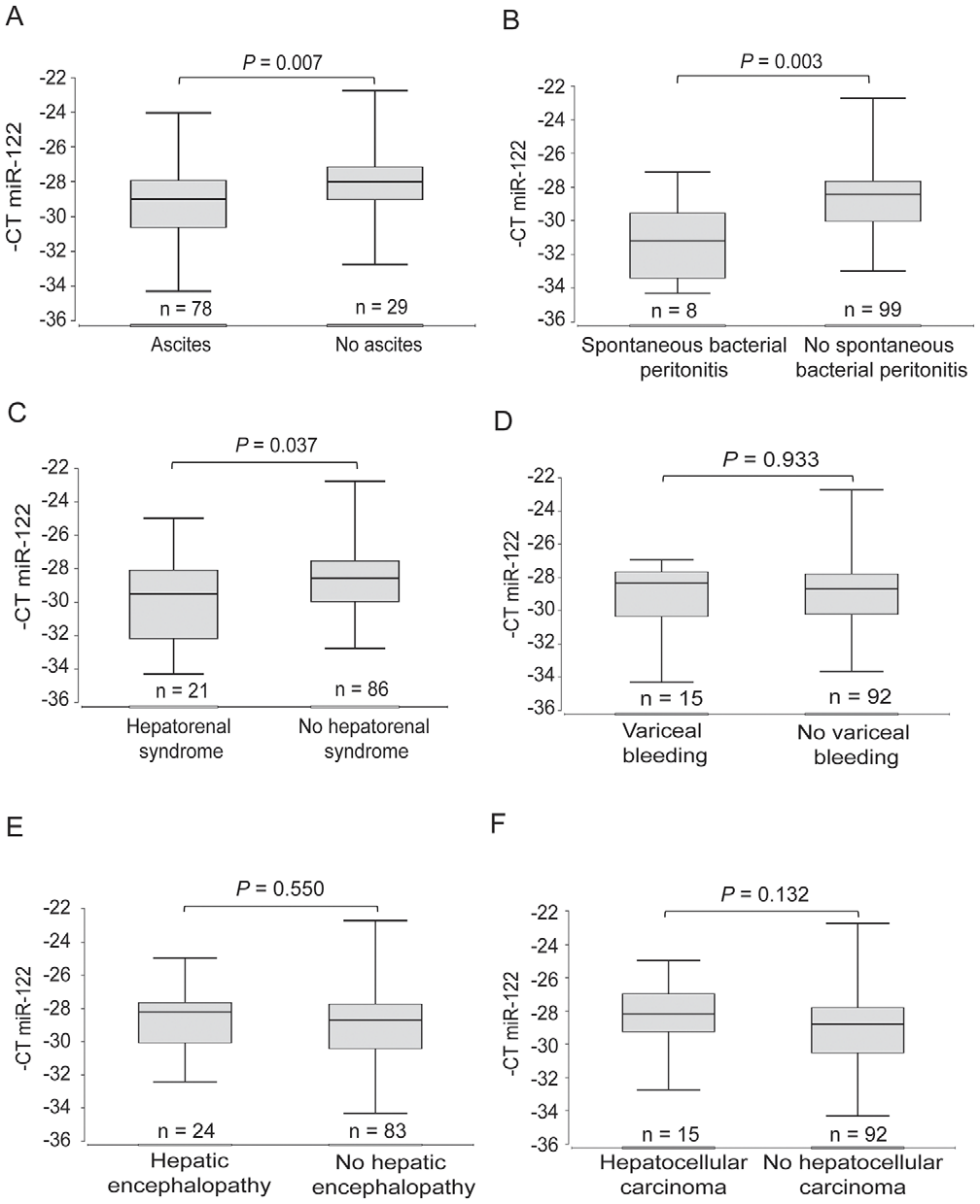
Serum miR-122 levels in complications of liver cirrhosis. The vertical lines indicate the range, the horizontal boundaries of the boxes represent the first and third quartile. The number of patients in each group is indicated in the figure.

## Methods

### Ethics statement

All patients gave their written informed consent. The study was approved by the Ethics Committee of the Goethe University Frankfurt, Germany. The study was performed in accordance with the 1975 Declaration of Helsinki and the REMARK (Reporting recommendations for tumor marker prognostic studies) guidelines.

**Table 2:** Correlation between miR-122 serum levels and laboratory parameters.

Parameter	Rank correlation coefficient (r)	*P*
Alanine aminotransferase (U/l)	0.402	<0.001
Aspartate aminotransferase (U/l)	0.358	<0.001
γ-glutamyltransferase (U/l)	0.365	<0.001
Alkaline phosphatase (U/l)	0.400	<0.001
Total serum protein (mg/dl)	0.071	0.465
Albumin (mg/dl)	0.069	0.481
International normalized ratio	−0.331	<0.001
Bilirubin (mg/dl)	−0.076	0.434
Creatinine (mg/dl)	−0.238	0.013
MELD^1^ score	−0.290	0.003

Abbreviation: ^1^Model of end stage liver disease.

### Selection of patients

Patients with liver cirrhosis who were admitted to our liver unit from May 2009 until May 2010 were prospectively enrolled into the present study. Inclusion criteria were liver cirrhosis assessed by histopathological examination or pathognomonic results in abdominal ultrasound examination, computer tomography or magnetic resonance imaging. Only in patients with inexplicit stages of fibrosis or unclear cause of liver disease biopsy was performed. In patients with characteristic sonomorphologic criteria of cirrhosis or decompensated liver disease no liver biopsy was performed. Exclusion criteria were an age below 18 years, former liver transplantation and a history of cancer within the last five years other than hepatocellular carcinoma. At the day of informed consent and enrollment of patients, blood samples were obtained for the analyses and were also tested for routine parameters of clinical chemistry. Furthermore, symptoms of hepatic decompensation (variceal bleeding, ascites, spontaneous bacterial peritonitis, hepatorenal syndrome or hepatic encephalopathy) were assessed. The primary end point was overall survival time. Death was recorded as event. The patients were followed-up until death, liver transplantation or last contact. Patients who underwent liver transplantation were excluded from the study from the day of transplantation. A flow chart showing the design of the study is shown in **Figure S1**.

**Figure 3:**
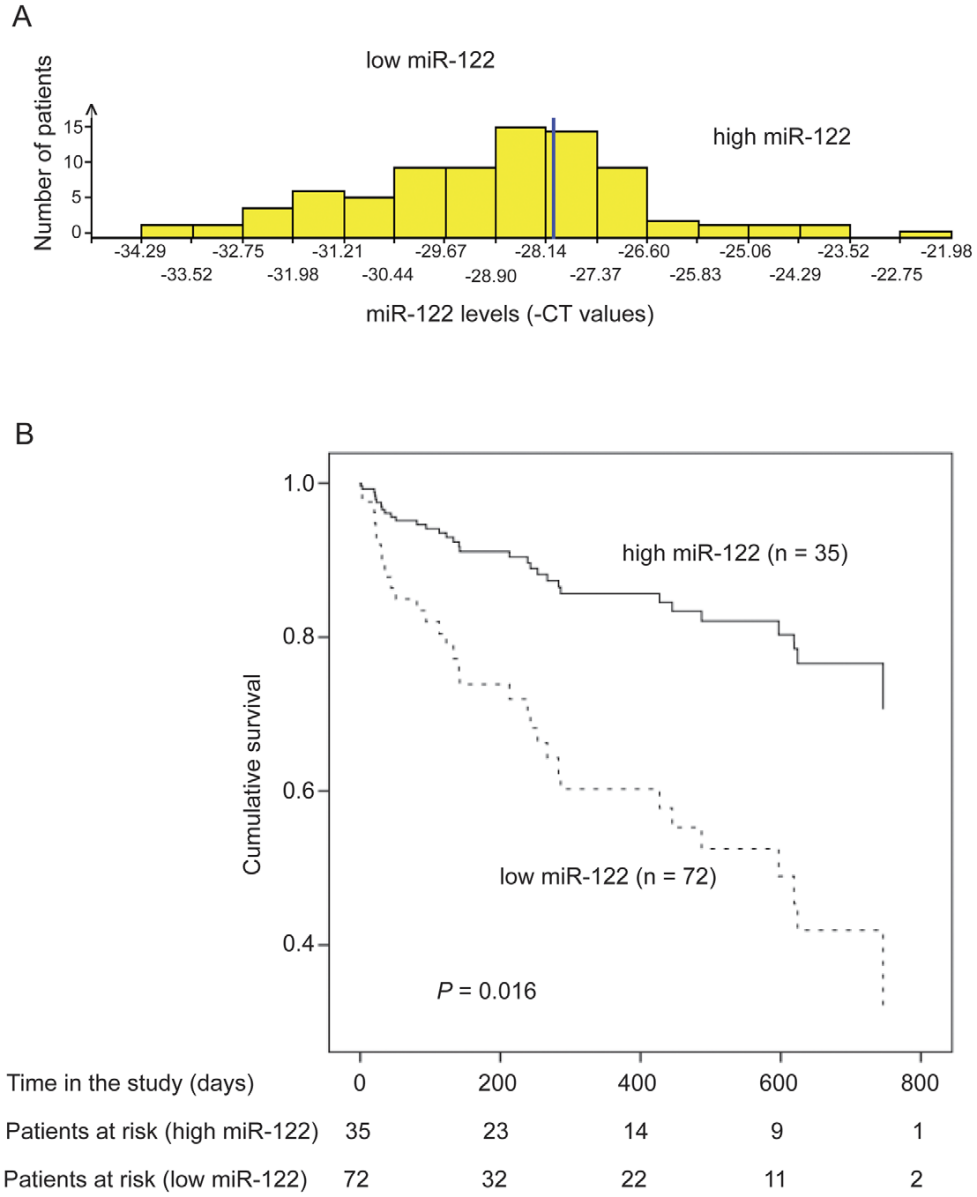
Serum miR-122 levels are associated with survival in patients with liver cirrhosis. (**A**) Distribution of serum miR-122 levels throughout the patients. The cut-off value for the patient cohorts is marked in blue. (**B**) Survival curves for patients with high or low serum miR-122 levels. The analysis was performed with the Cox regression model.

### Validation cohort

The validation cohort consisted of a second cohort of cirrhotic patients who were admitted to our hospital from October 2010 until May 2011 and were prospectively enrolled. Inclusion and exclusion criteria were the same as in the test cohort. Follow-up and assessment of overall survival were performed as in the test cohort. All patients gave their written informed consent to participate in the study.

**Table 3:** Univariate and multivariate analysis of parameters associated with overall survival.

	Univariate analysis			Multivariate analysis		
Parameter	HR	95% CI	*P* value	HR	95% CI	*P* value
low miR-122	3.259	1.252–8.484	0.016	3.405	1.295–8.951	0.013
MELD <18	0.262	0.126–0.545	<0.001	0.212	0.098–0.460	<0.001
Male Sex	0.521	0.257–1.054	0.070	0.353	0.170–0.734	0.005
Age <65 years	0.717	0.356–1.446	0.353			

Abbreviations:

HR, hazard ratio; CI, confidence interval; MELD, model of end stage liver disease.

### Blood sampling

Peripheral blood was collected from each individual at the day of enrollment into the study. The serum tubes were centrifuged at 1500× g for 10 min at 4°C, followed by an additional centrifugation step at 2000× g 4°C to completely remove any remaining cells. The serum samples were aliquoted and stored at −80°C until further use.

**Figure 4:**
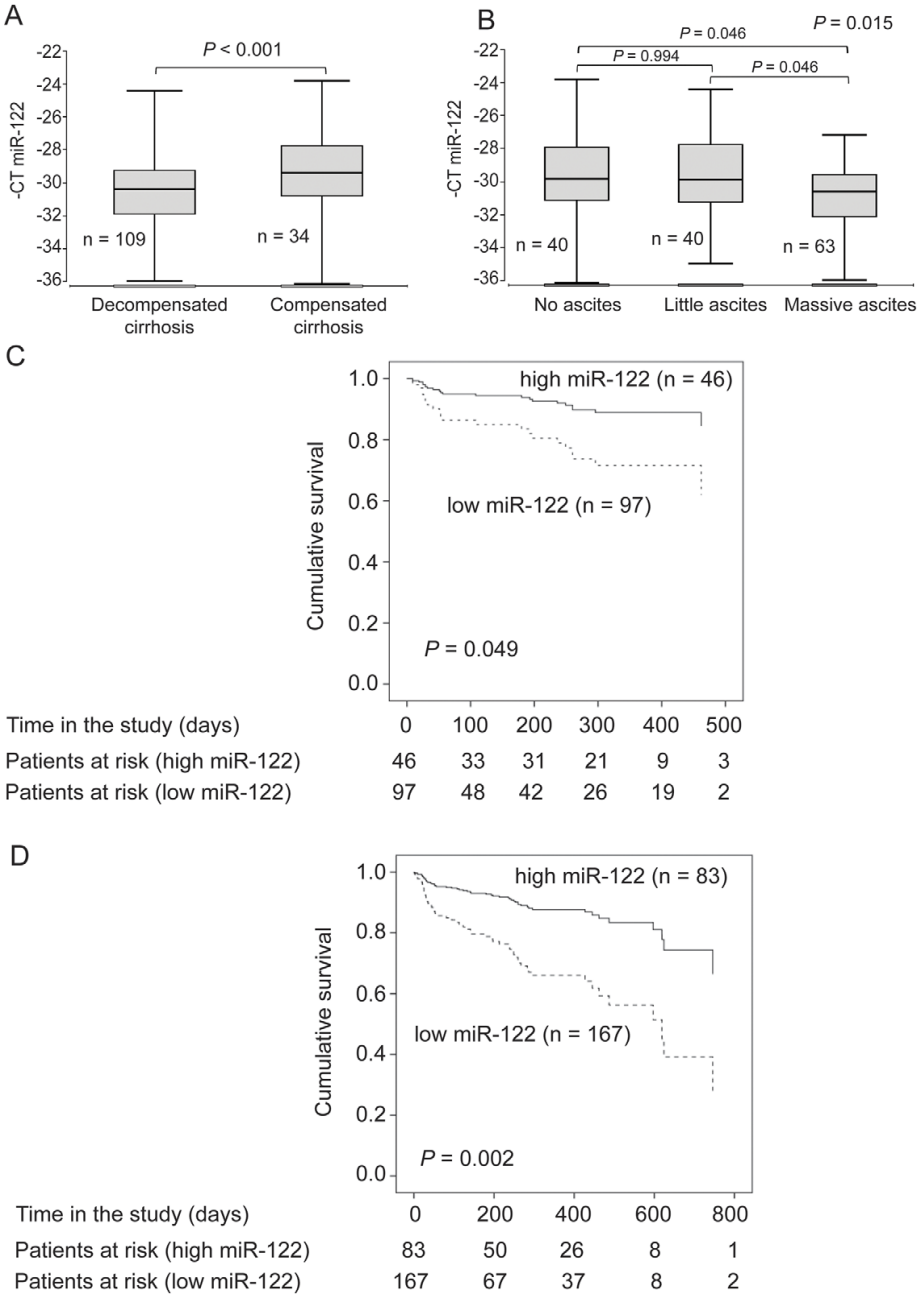
Serum miR-122 levels in the validation cohort and overall survival in the combined cohort. (**A**) Serum miR-122 in patients with and without hepatic decompensation. (**B**) miR-122 levels in patients with and without ascites. The vertical lines indicate the range, the horizontal boundaries of the boxes represent the first and third quartile. The analysis was performed with Kruskal-Wallis test and the Bonferroni correction was used for the sub-analysis. The number of patients in each group is indicated in the figure. (**C**) Survival curves for patients in the validation cohort with high or low serum miR-122 levels. The analysis was performed with the Cox regression model. (**D**) Survival curves for patients in the combined cohort with high or low serum miR-122 levels. The analysis was performed with the Cox regression model.

### Clinical chemistry

Standard parameters of liver and kidney function were measured at the central laboratory of the Frankfurt University Hospital.

### Detection of miRs by quantitative real-time reverse-transcription (RT)-PCR

Isolation of serum RNA and quantification of the miR-122 levels were performed as described previously [24], [25]. Total RNA was extracted from 500 µl of serum with the miRVana RNA isolation kit and TriReagentLS (Sigma-Aldrich, St. Louis, MO) and chloroform according to the *mir*Vana^TM^ miRNA isolation kit protocol. cDNA was reverse transcribed from 5 µl of RNA with the TaqMan miRNA reverse transcription kit. miR-122 levels were assessed by a TaqMan miRNA assay specific for miR-122 from Applied Biosystems (ABI, Foster City, CA) on a StepOne^TM^Plus Real-Time PCR System (ABI, Foster City, CA). Quantitative real-time PCRs were performed in duplicates and means were calculated. The results of quantitative PCR values were given as negatives of the C_T_ (cycle threshold) values which correlate with the amount of miRs in the serum.

### Statistical analysis

Data were analyzed using the BiAS software for windows (version 9.11, Epsilon-Verlag, Darmstadt, Germany) and SPSS version 20 (IBM, Chicago, IL). Death was considered as event. Follow-up time was time until death or last contact to patient. In patients who underwent liver transplantation the follow-up was stopped at the day of liver transplantation. From the date of transplantation the patients were excluded from further analysis. Overall survival rates were assessed for all patients using the Cox regression model. Survival curves were calculated with the Cox model. Independent predictors of survival were determined with a multivariate Cox regression analysis using forward stepwise (likelihood ratio) entry. The nonparametric Wilcoxon-Mann-Whitney and Kruskal-Wallis tests were used to determine differences between groups of patients. A Bonferroni correction was used when multiple subgroup comparisons were performed. *P* values <0.05 were considered to be significant. In the box plots the vertical lines indicate the range, the horizontal boundaries of the boxes represent the first and third quartile. The correlation coefficients r were calculated by using the Spearman correlation.

## Results

From May 2009 until May 2010 107 patients with liver cirrhosis who were admitted to our liver unit at the Goethe University Hospital were prospectively enrolled into the present study. The patient characteristics are shown in **Table** **1**. 90 patients (84.1%) showed signs of hepatic decompensation (variceal bleeding, ascites, hepatic encephalopathy, hepatorenal syndrome or spontaneous bacterial peritonitis), whereas 17 patients had compensated liver cirrhosis. Some patients had more than one etiology of chronic liver disease. The mean duration (± SD) of follow-up was 220±267 days. 26 of the 107 patients died within the study time. 14 patients underwent liver transplantation at the Goethe University Hospital Frankfurt. The median MELD score at study entry in the cohort of patients was 16.

### Low serum miR-122 levels are associated with decompensation of liver cirrhosis

miR-122 levels in sera of patients with and without hepatic decompensation were assessed. Serum levels of miR-122 in patients with hepatic decompensation were significantly lower compared to patients with compensated liver disease (*P* = 0.012) (**Figure** **1**). To assess if reduced miR-122 serum levels were associated with complications of liver cirrhosis, the miR-122 serum levels were examined in patients with and without ascites, spontaneous bacterial peritonitis, hepatorenal syndrome, variceal bleeding, hepatic encephalopathy and hepatocellular carcinoma. As shown in **Figure** **2A–C**, patients with ascites (*P* = 0.007), spontaneous bacterial peritonitis (*P* = 0.003) or hepatorenal syndrome (*P* = 0.037) had significantly lower serum levels of miR-122 than patients without the respective complication. In contrast, no significant differences were observed between patients with and without variceal bleeding (*P* = 0.933), hepatic encephalopathy (*P* = 0.550) or hepatocellular carcinoma (*P* = 0.132) (**Figure** **2D–F**).

### Serum miR-122 levels correlate with parameters of liver damage and hepatic functional capacity

Serum/plasma levels of miR-122 correlate with hepatic necroinflammation, liver damage and aminotransferase levels in acute and chronic liver diseases [22]–[26]. Therefore, the relation between serum miR-122 levels and standard laboratory parameters in the cohort of patients with liver cirrhosis were investigated. The results are displayed in **Table** **2**. Highly significant correlations were found between serum miR-122 levels and alanine aminotransferase (ALT), aspartate aminotransferase (AST), γ-glutamyltransferase (GGT) and alkaline phosphatase (ALP), i. e. parameters of liver cell damage. Moreover, there was a negative correlation between serum miR-122 levels and the international normalized ratio (INR), an indicator of liver function. In contrast, there were no significant relations between miR-122 serum levels and the serum bilirubin, serum albumin and total serum protein levels. There was a negative correlation between the serum levels of miR-122 and creatinine, i. e. patients with higher serum creatinine had lower miR-122 serum levels. Finally, a significant correlation between the MELD score and miR-122 levels were observed.

### The serum miR-122 level is a marker for mortality in patients with liver cirrhosis

The finding that patients with hepatic decompensation, who have a worse prognosis than patients with compensated liver cirrhosis, had lower miR-122 serum levels than patients with compensated liver disease, led to the assumption that miR-122 might be of prognostic relevance in cirrhotic patients. Therefore, the patients were grouped according to their miR-122 serum concentration in tertiles. The distribution of serum miR-122 levels among the patients is shown in **Figure** **3A**. The third of patients with the highest miR-122 levels was compared to the two thirds of patients with lower miR-122 serum concentration and a univariate Cox regression analysis was performed. There was a significant association between miR-122 levels and overall survival, i. e. patients with low miR-122 levels showed shorter survival times (*P* = 0.016, hazard ratio 3.259, 95% confidence interval 1.252–8.484). Survival curves illustrate the different survival times between patients with low and high serum miR-122 concentrations (**Figure** **3B**).

To investigate if the miR-122 serum levels might be associated with overall survival independently from the MELD score, an age and sex adjusted multivariate Cox regression analysis using forward stepwise (likelihood ratio) was performed. As shown in **Table** **3**, a low MELD score (*P*<0.001), male sex (*P* = 0.005) and low miR-122 serum levels (*P* = 0.013) were independently associated with survival.

### Validation of results in a second cohort of patients

To confirm the finding that miR-122 serum levels are lower in patients with hepatic decompensation and are associated with overall survival, a second cohort of cirrhotic patients was analyzed. 143 patients with liver cirrhosis were prospectively enrolled from October 2010 until May 2011 at the Liver Department of the Goethe University Hospital. Patients' characteristics are shown in **Table** **1**. The median follow-up time was 232±172 days. 21 patients died within the observation time and 13 patients underwent liver transplantation. 109 of the 143 patients (76.2%) showed decompensated liver disease.

Similar to the test cohort, in the validation cohort lower levels of miR-122 were found in patients with hepatic decompensation (*P* = 0.026) (**Figure** **4A**) and in patients with ascites (*P* = 0.015) (**Figure** **4B**). Patients with lower serum miR-122 levels had also a shorter overall survival time (*P* = 0.049, hazard ratio 2.683, 95% confidence interval 1.294–5.564) (**Figure** **4C**).

Finally, the data of the two independent cohorts were combined and univariate and multivariate sex and age adjusted Cox regression with inclusion of the MELD score were performed. In the univariate as well as in the multivariate analysis miR-122 was significantly associated with overall survival (*P* = 0.002, hazard ratio 3.161, 95% confidence interval 1.539–6.494 and *P* = 0.005, hazard ratio 2.872, 95% confidence interval 1.371–6.014, respectively). The overall survival curves for all patients according to higher and lower miR-122 serum concentrations are shown in **Figure** **4D**.

## Discussion

MicroRNAs in serum or plasma are increasingly recognized as new potentially useful disease parameters readily accessible in the blood. Although several studies have investigated the role of miRs in liver diseases, prospective studies examining the role of serum or plasma miRNAs in human diseases are still rare. Due to its almost exclusive expression in the liver, the presence of miR-122 in serum is highly indicative for liver processes [15], [16]. In this prospective clinical cohort study we evaluated if the serum levels of miR-122 might be a suitable parameter for disease severity and prognosis in patients with liver cirrhosis. Our data obtained in two independent patient cohorts suggest that serum miR-122 is an indicator for impaired liver function and independent new parameter for survival of patients with liver cirrhosis. The miR-122 levels were significantly lower in patients with hepatic decompensation. Categorization of the patients in groups of low and high miR-122 serum concentration revealed that patients with low miR-122 serum levels had shorter survival times than patients with higher serum miR-122 levels. Thus, the serum miR-122 level might be a useful prognostic parameter in patients with liver cirrhosis, supplementing the MELD score in assessing the risk of death. In fact, the multivariate Cox regression analysis revealed that the MELD score and the serum miR-122 level were independently associated with the survival time, suggesting that the serum miR-122 level provides additional prognostic information to the MELD score. Similarly, in the validation cohort lower miR-122 serum levels were found in patients with decompensated liver disease confirming the initial results and suggesting that serum miR-122 levels are indicators of hepatic functional capacity. Combining the overall survival data of both cohorts lead to a highly significant difference concerning overall survival between patients with higher and lower miR-122 serum concentrations. The suggestion that miR-122 levels are associated with hepatic functional capacity is supported by our findings that low serum miR-122 levels were associated with the presence of hepatorenal syndrome, ascites and spontaneous bacterial peritonitis, all of which are associated with shorter survival of patients.

Similar to previous studies in patients with chronic or acute liver damage [22]–[27], we found a positive correlation between the serum level of miR-122 and serum aminotransferases, indicating that miR-122 represents at least in part ongoing liver damage and cell death. In addition, there was a strong negative correlation between serum miR-122 levels and INR in our patients with liver cirrhosis, which was not observed in patients with HCV-induced chronic hepatitis [22]. It can be anticipated that the release of miR-122 from necrotic hepatocytes, secretion of miR-122 from hepatocytes within nanovesicles [28] and reduced degradation are the major sources of extracellularly circulating miR-122. Furthermore, distribution may also be a relevant factor. If release from necrotic hepatocytes or reduced degradation were the major source of serum miR-122, a positive correlation between hepatic complications and miR-122 should be expected. In the present study, however, we observed lower levels of miR-122 in patients with more advanced disease, indicating that reduced serum miR-122 is most likely the result of reduced release from heptocytes. Another possibility is that miR-122 serum levels are reduced due to higher volume distribution in patients with ascites. This indicates that in patients with liver cirrhosis, the miR-122 serum level might be a marker for hepatic functional capacity, whereas at earlier stages of liver disease, the serum miR-122 level is mainly an indicator of necroinflammatory activity and cell death in the liver. As release from damaged hepatocytes might be the major source of hepatocyte-derived miRs [19], it is conceivable that in cirrhotic patients who lost a big proportion of hepatocytes and thus have less functional hepatic capacity, the release of miRs upon damage might be lower than in patients with higher amounts of healthy liver tissue.

An additional finding of the present study is the inverse correlation between the serum concentrations of miR-122 and creatinine. Patients with hepatorenal syndrome had highly significant lower miR-122 serum levels than patients without this complication. Different from the present study in a recent publication no correlation between serum miR-122 and creatinine levels was found in patients with toxic liver damage [22]. The differences in these findings are probably due to the different pathogenesis of liver failure and concomitant kidney failure between acute-on-chronic decompensation in our patients and acute drug-induced liver failure in the other cohort [22]. In our patients low miR-122 serum levels were correlated with deterioration of liver capacity which is accompanied by kidney failure, whereas in acute liver damage, enhanced miR-122 levels represent hepatocyte damage and death [21], [22]. However, we did not find any correlation between the serum levels of miR-122 and albumin, total protein or bilirubin. One explanation for this might be that patients with heavily impaired liver function often receive parenteral albumin substitution which adulterates the measured protein serum levels. Furthermore, bilirubin and albumin levels may vary depending on nutrition status, drug use or cholestasis and are biased by different half-life times and thus are not the most reliable indicators of liver function [29].

In summary, the data of the present study indicate that the serum level of the hepatocyte specific miR-122 is reduced in patients with hepatic decompensation, and that lower miR-122 levels are associated with ascites, spontaneous bacterial peritonitis and hepatorenal syndrome. Lower miR-122 concentrations were associated with mortality independently from the MELD score. Therefore, serum miR-122 is a new potential parameter for liver function and a prognostic parameter in patients with liver cirrhosis.

## Supporting Information

Figure S1 **Design of the study.**
(TIF)

## References

[1] Cárdenas A, Ginès P (2011) Management of patients with cirrhosis awaiting liver transplantation. Gut 60: 412–421. 21193458 10.1136/gut.2009.179937

[2] European Association for the Study of the Liver (2010) EASL clinical practice guidelines on the management of ascites, spontaneous bacterial peritonitis, and hepatorenal syndrome in cirrhosis. J Hepatol 53: 397–417. 20633946 10.1016/j.jhep.2010.05.004

[3] Garcia-Tsao G, Bosch J (2010) Management of varices and variceal hemorrhage in cirrhosis. N Engl J Med 362: 823–832. 20200386 10.1056/NEJMra0901512

[4] Wiesner RH (2005) Patient selection in an era of donor liver shortage: Current US policy. Nat Clin Pract Gastroenterol Hepatol 2: 24–30. 16265097 10.1038/ncpgasthep0070

[5] Malinchoc M, Kamath PS, Gordon FD, Peine CJ, Rank J, et al (2000) A model to predict poor survival in patients undergoing transjugular intrahepatic portosystemic shunts. Hepatology 31: 864–871. 10733541 10.1053/he.2000.5852

[6] Kamath PS, Wiesner RH, Malinchoc M, Kremers W, Therneau TM, et al (2001) A model to predict survival in patients with end stage liver disease. Hepatology 33: 464–470. 11172350 10.1053/jhep.2001.22172

[7] Kamath PS, Kim WR (2007) The Model for End stage Liver Disease (MELD). Hepatology 45: 797–805. 17326206 10.1002/hep.21563

[8] Alessandria C, Ozdogan O, Guevara M, Restuccia T, Jiménez W, et al (2005) MELD score and clinical type predict prognosis in hepatorenal syndrome: relevance to liver transplantation. Hepatology 41: 1282–1289. 15834937 10.1002/hep.20687

[9] Bambha K, Kim WR, Pedersen R, Bida JP, Kremers WK, et al (2008) Predictors of early re-bleeding and mortality after acute variceal haemorrhage in patients with cirrhosis. Gut 57: 814–820. 18250126 10.1136/gut.2007.137489

[10] Ambros V (2004) The functions of animal microRNAs. Nature 431: 350–355. 15372042 10.1038/nature02871

[11] Kloosterman WP, Plasterk RH (2006) The diverse functions of microRNAs in animal development and disease. Dev Cell 11: 441–450. 17011485 10.1016/j.devcel.2006.09.009

[12] Chen X, Ba Y, Ma L, Cai X, Yin Y, et al (2008) Characterization of microRNAs in serum: a novel class of biomarkers for diagnosis of cancer and other diseases. Cell Res 18: 997–1006. 18766170 10.1038/cr.2008.282

[13] Mitchell PS, Parkin RK, Kroh EM, Fritz BR, Wyman SK, et al (2008) Circulating microRNAs as stable bloodbased markers for cancer detection. Proc Natl Acad Sci USA 105: 10513–10518. 18663219 10.1073/pnas.0804549105 PMC2492472

[14] Esquela-Kerscher A, Slack FJ (2006) Oncomirs – microRNAs with a role in cancer. Nat Rev Cancer 6: 259–269. 16557279 10.1038/nrc1840

[15] Lagos-Quintana M, Rauhut R, Yalcin A, Meyer J, Lendeckel W, et al (2002) Identification of tissue-specific microRNAs from mouse. Curr Biol 12: 735–739. 12007417 10.1016/s0960-9822(02)00809-6

[16] Chang J, Nicolas E, Marks D, Sander C, Lerro A, et al (2004) miR-122, a mammalian liver-specific microRNA, is processed from hcr mRNA and may downregulate the high affinity cationic amino acid transporter CAT-1. RNA Biol 1: 106–113. 17179747 10.4161/rna.1.2.1066

[17] Boutz DR, Collins PJ, Suresh U, Lu M, Ramírez CM, et al (2011) Two-tiered approach identifies a network of cancer and liver disease-related genes regulated by miR-122. J Biol Chem. 286: 18066–18078. 10.1074/jbc.M110.196451 PMC3093880 21402708

[18] Coulouarn C, Factor VM, Andersen JB, Durkin ME, Thorgeirsson SS (2009) Loss of miR-122 expression in liver cancer correlates with suppression of the hepatic phenotype and gain of metastatic properties. Oncogene. 28: 3526–3536. 10.1038/onc.2009.211 PMC3492882 19617899

[19] Wang K, Zhang S, Weber J, Baxter D, Galas DJ (2010) Export of microRNAs and microRNA-protective protein by mammalian cells. Nucl Acids Res 38: 7248–7259. 20615901 10.1093/nar/gkq601 PMC2978372

[20] Wang K, Zhang S, Marzolf B, Troisch P, Brightman A, et al (2009) Circulating microRNAs, potential biomarkers for drug-induced liver injury. Proc Natl Acad Sci USA 106: 4402–4407. 19246379 10.1073/pnas.0813371106 PMC2657429

[21] Laterza OF, Lim L, Garrett-Engele PW, Vlasakova K, Muniappa N, et al (2009) Plasma microRNAs as sensitive and specific biomarkers of tissue injury. Clin Chem 55: 1977–1983. 19745058 10.1373/clinchem.2009.131797

[22] Starkey Lewis PJ, Dear J, Platt V, Simpson KJ, Craig DG, et al (2011) Circulating microRNAs as potential markers of human drug induced liver injury. Hepatology 5: 1767–1776. 10.1002/hep.24538 22045675

[23] Zhang Y, Jia Y, Zheng R, Guo Y, Wang Y, et al (2010) Plasma microRNA-122 as a biomarker for viral-, alcohol-, and chemical-related hepatic diseases. Clin Chem 56: 1830–1838. 20930130 10.1373/clinchem.2010.147850

[24] Bihrer V, Friedrich-Rust M, Kronenberger B, Forestier N, Haupenthal J, et al (2011) Serum miR-122 as a biomarker of necroinflammation in patients with chronic hepatitis C virus infection. Am J Gastroenterol 106: 1663–1669. 21606975 10.1038/ajg.2011.161

[25] Waidmann O, Bihrer V, Pleli T, Farnik H, Berger A, et al (2012) Serum microRNA 122 levels in different groups of patients with chronic hepatitis B virus infection. J Viral Hepat 19: e58–65. 22239527 10.1111/j.1365-2893.2011.01536.x

[26] Xu J, Wu C, Che X, Wang L, Yu D, et al (2011) Circulating microRNAs, miR-21, miR-122, and miR-223, in patients with hepatocellular carcinoma or chronic hepatitis. Mol Carcinog. 50: 136–142. 10.1002/mc.20712 21229610

[27] Farid WR, Pan Q, van der Meer AJ, de Ruiter PE, Ramakrishnaiah V, et al (2012) Hepatocyte-derived micrornas as serum biomarker of hepatic injury and rejection after liver transplantation. Liver Transpl 18: 290–297. 21932376 10.1002/lt.22438

[28] Kogure T, Lin WL, Yan IK, Braconi C, Patel T (2011) Intercellular nanovesicle-mediated microRNA transfer: a mechanism of environmental modulation of hepatocellular cancer cell growth. Hepatology. 54: 1237–1248. 10.1002/hep.24504 PMC3310362 21721029

[29] Jalan R, Hayes PC (1995) Review article: quantitative tests of liver function. Aliment Pharmacol Ther 9: 263–270. 7654888 10.1111/j.1365-2036.1995.tb00380.x

